# V30 as a predictor for radiation-induced hypothyroidism: a dosimetric analysis in patients who received radiotherapy to the neck

**DOI:** 10.1186/1748-717X-9-104

**Published:** 2014-05-02

**Authors:** Zuleyha Akgun, Beste M Atasoy, Zeynep Ozen, Dilek Yavuz, Bahadir Gulluoglu, Meric Sengoz, Ufuk Abacioglu

**Affiliations:** 1 Department of Radiation Oncology, Bezmi Alem Vakif University Medical School, Adnan Menderes Bulvari, 34093 Istanbul, Fatih, Turkey; 2 Department of Radiation Oncology, School of Medicine, Marmara University, Istanbul, Turkey; 3 Neolife Cancer Center Radiation Oncology Clinic, Istanbul, Turkey; 4 Department of Internal Medicine Division of Endocrinology, School of Medicine, Marmara University, Istanbul, Turkey; 5 Department of General Surgery, School of Medicine, Marmara University, Istanbul, Turkey; 6 Acibadem University Kozyatagi Hospital Radiation Oncology Clinic, Istanbul, Turkey

**Keywords:** Dosimetric analysis, Hypothyroidism, Radiotherapy

## Abstract

**Introduction:**

The purpose of this study is to evaluate the possible predictors of thyroid disorders after neck radiotherapy, with a focus on radiation dose-volume factors.

**Methods:**

Thyroid function was measured in 100 patients who had received radiotherapy to the neck, including the thyroid. All radiation-induced thyroid dysfunctions were determined with an endpoint of abnormal thyroid stimulating hormone (TSH), free triiodothyronine (fT3) and thyroxine (fT4) and thyroid peroxidase antibodies and (TPA). The total volume of the thyroid, mean radiation dose to the thyroid (Dmean) and thyroid volume percentage that received radiation doses of 10–50 Gy (V10-V50) were calculated in all patients. The evaluated risk factors for thyroid dysfunction included dose-volume parameters, sex, age, previous surgery, chemotherapy and comorbidity.

**Results:**

There were 52 patients with hypothyroidism and V30 (p = 0.03), thyroid volume (p = 0.01) and Dmean (p = 0.03) appeared to be correlated with hypothyroidism in univariate analysis. However, there was not association found in multivariate analysis for these factors.

**Conclusions:**

Thyroid disorders after radiation therapy to the neck still represent a clinically underestimated problem. V30 may be a useful tool for evaluating the risk of hypothyroidism when determining an individual patient’s treatment.

## Background

The thyroid gland is one the most radiosensitive normal tissues in the human body. It is frequently affected by radiotherapy in head and neck patients, and thyroid dysfunctions are commonly seen following irradiation [1,2]. The incidence of radiation-induced hypothyroidism has been described mostly within the range of 20–30% and the reports are currently increasing in literature [1-25]. Nonetheless, it still remains an underestimated problem in routine clinical practice [3-5]. Some dosimetric studies exploring radiation-induced thyroid dysfunction have suggested that a thyroid dose of ≥30 Gy is associated with higher risk for hypothyroidism [1,4,6,7]. However, Kim et al. [8] found that the percentage of thyroid volume absorbing >45 Gy was observed to be the optimal variable for predicting the development of hypothyroidism, and they suggested a V45 of 50% as a possible dose-volumetric threshold for radiation-induced hypothyroidism.

In this study, we aimed to evaluate the incidence of thyroid dysfunction following radiotherapy and to explore any potential correlation between thyroid dysfunction and radiation doses in our patients.

## Methods

This study was approved by the Ethical Committee of Marmara University School of Medicine. Between May 2005 and March 2010, 106 patients who had histologically proven non-thyroid malignancies located in the head and neck region who were treated in our center were recruited for the analysis. The patients who had existing thyroid dysfunction (n = 6) before radiotherapy were excluded from the analysis. There were 30 patients who had surgery to primary tumor and/or neck before radiotherapy. All patients were irradiated at the primary site and the neck and were regularly followed after radiotherapy. The patients and treatment characteristics are summarized in Table 1.

**Table 1:** Characteristics of the study group

**Characteristics**	**n**
**Gender**	
Female	32
Male	68
**Age (years)**	
Median	50
Range	17- 82
**Tumor site**	
Nasopharynx	38
Larynx & hypopharynx	35
Oral cavity & oropharynx	15
Lymphoma	12
**Chemotherapy**	
Sequential	30
Taxane 75 mg/m^2^/21 d and cisplatin 75 mg/m^2^/21 d	
Concomitant	31
Cisplatin 75 mg/m^2^/21 d	
Previous surgery to primary tumor side and/or neck	30
**Comorbidity (diabetes and/or hypertension)**	17

### Radiotherapy

All patients underwent our center’s routine procedure for head and neck tumor patients. The patients first underwent a simulation using immobilization with a thermoplastic mask to ensure a reproducible set-up. The patients then underwent a treatment-planning computed tomography (CT) scan with images obtained at 3- to 5-mm slice intervals. The data were transferred to the planning system and 3-D treatment planning using Eclipse (Varian Medical Systems, Palo Alto, CA, USA) was performed for all the patients. Either two opposite equally weighted lateral fields plus one anterior field or anterior-posterior fields or five fields, were used. Treatment was performed with photons (6 MV or 18 MV) and/or electrons (6 MeV, 9 MeV to 12 MeV) using a linear accelerator (Saturne 42, 800 series, General Electric, Buc, France). A total median dose of 60 Gy (range, 36–70 Gy) in 20 to 35 daily fractions of 1.8 or 2 Gy was delivered to the target.

### Dosimetric analysis

Treatment plans from all patients were included in this study and the thyroid gland was contoured on each individual CT-slice by the same Radiation Oncologist (ZA). The minimum (Dmin), the maximum (Dmax) and the mean (Dmean) doses the thyroid gland received and the percentage of thyroid volume receiving 10 Gy (V10), 20 Gy (V20), 30 Gy (V30), 40 Gy (V40) and 50 Gy (V50) were calculated using dose volume histograms.

### Thyroid function assessments

The patients were followed for thyroid functions after the completion of radiotherapy with periodic thyroid stimulant hormone (TSH), free thyroxin (fT4), free triiodothyronine (fT3) and thyroid peroxidase antibody (TPA) tests. The first test was obtained at 3 to 6 months after radiotherapy, with the next test at a six months interval thereafter. All tests were measured in the same laboratory using electrochemiluminescence methods (Roche Elecsys 2010 immunoassay analyzer, Roche Diagnostics GmbH, Mannheim, Germany). According to recommendations from the American Thyroid Association, hypothyroidism was defined as a TSH value greater than the upper limit of the normal range. The upper cut off range for TSH that was used was 4 mIU/L for individuals under 60 years old, 6 mIU/L for those in the 7^th^ decade and 7 for those in the 8^th^ decade [9].

### Statistical analysis

Statistical analyses were performed using NCSS 2007 & PASS 2008 Statistical Software (Utah, USA). For statistical analysis, we evaluated the following variables: thyroid absorbed dose, thyroid volume, previous surgery to the primary tumor or to neck, chemotherapy, comorbidity, age, and sex. The relationship between any clinical or treatment parameter and the incidence of thyroid function disorders were analyzed using the appropriate chi-square test or Fisher’s exact test for categorical variables and the nonparametric Mann–Whitney U test for continuous variables. The median and range were used to describe all continuous variables. The chi-square test was used to assess the data for correlations among the dose-volume parameters. Continuous variables were dichotomized at median (range) values. A multivariate was used (logistic regression analysis with backward variable selection) and β value were calculated for the variables that found significant in univariate analysis. P < 0.05 were regarded as significant.

## Results

The median follow-up was 47 months (range, 11 to 80 months). There were 52 patients in whom previous thyroid function tests had been reached. All thyroid function test results were in normal range and none of the patients has received a levothyroxine treatment before radiotherapy. During the follow-up, 52 patients developed clinical hypothyroidism and they all were treated with levothyroxine. There were 12 patients who developed thyroiditis, as determined by thyroid ultrasound findings. There were no cases of hypothyroidism (in which the low level of TSH is associated with low levels of fT3 and/or fT4) or hyperthyroidism (in which the high level of TSH is associated with high levels of fT3 and/or fT4) related to pituitary gland dysfunction. The patients’ distribution in numbers according to the time that hypothyroidism was detected is shown in Table 2. In summary, the incidence of first diagnosis of hypothyroidism was highest in the first year, especially in the second 6^th^ month period.

**Table 2:** The number of patients developed hypothyroidism according to month intervals

**Interval (months)**	**Number of patients**
2-6	10
7-12	13
13-18	2
19-24	5
25-30	7
31-36	6
37-42	2
43-48	3
49-54	1
55-60	2
61-66	1
67-72	1

The univariate analysis for clinical and dosimetric parameters are shown in Table 3. There were no correlations observed between the incidence of hypothyroidism and the patients’ comorbidity, previous surgery, chemotherapy, age and gender. Additionally, there were no significant relationships observed between Dmax or Dmin and hypothyroidism. However, Dmean (p = 0.03), the thyroid volume (31.59 cc vs. 14.25 cc; p = 0.01) and the analysis of irradiated thyroid volume threshold showed that having whole of thyroid volume irradiated to 30 Gy was a statistically significant predictor for hypothyroidism (p = 0.03). Meanwhile, V10, V20, V40 or V50 were not found to be associated with higher risks of thyroid disorder. In multivariate analysis there was no correlation found for significant parameters of Vmean (p = 0.627, β = −0.102), thyroid volume (p = 0.06, β = −0.188) or V30 (p = 0.07, β = 0.181).

**Table 3:** Clinical and dosemiteric factors in patients with and without hypothyroidism

	**Normal**	**Hypothyroidism**	***P***
	**(n = 48)**	**(n = 52)**	
Median Age (year)	53	48	0.23
Comorbidity	9	8	0.30
Surgery	16	14	0.26
Chemotherapy	32	29	0.22
Gender			0.12
Female	46%	54%	
Male	60%	40%	
	**Median (range)**		
Thyroid volume	31.59 (5.7–99)	14.25 (0.6–69.6)	0.01
Dmin (≥40.1 Gy)	48.1 (0–61.0)	52.1 (0.6–59.7)	0.42
Dmax (≥57.6 Gy)	50 (19.9–75.3)	47.9 (19.4–71.0)	0.49
Dmean (≥47.3 Gy)	44.2 (5.7–67.3)	56.2 (3.8–66.5)	0.03
V10 (≥100%)	75.0 (13–100)	89.1 (9–100)	0.06
V20 (≥100%)	67.5 (7–100)	82.6 (0–100)	0.06
V30 (≥100%)	59.6 (0–100)	78.3 (0–100)	0.03
V40 (≥94%)	45.9 (0–100)	53.6 (0–100)	0.36
V50 (≥44%)	54.8 (0–100)	45.2 (0–100)	0.06

## Discussions

The thresholds for direct thyroid radiation damage are not clear. Investigators have previously reported that the incidence of hypothyroidism in various series of patients treated for head and neck cancer varies in a wide range [1-25]. However, most reports state the incidence to be between 20% and 53% and the incidence of 52% found in our study is consistent with these findings. Several dosimetric studies about radiotherapy related thyroid dysfunction and dose volume distribution have been performed in patients receiving irradiation to the thyroid gland [5,7,8,10,11]. Yoden et al. [14] reported that the correlation between the percentage of the volume receiving 10 Gy (V10), 20 Gy (V20) and 30 Gy (V30) is a possible predictor of hypothyroidism. Additionally, Bhandare et al. [5] reported a possible dose-volume effect on the increase in the incidence of hypothyroidism, with an increased incidence when 50% of the thyroid volume received greater radiation doses. Our data showed that V30 was a statistically significant predictor of the development of hypothyroidism (p = 0.03). In our study, the thyoid gland was an organ at risk in the treatment field instead of being a target tissue received high doses. There were fewer patients that the gland received high doses like primary tumor (≥40 Gy) and this may be the reason the insignificant correlation for V40 and V50.

Jerezeck et al. [4] showed that smaller thyroid volume was associated with a higher incidence of thyroid toxicity, and Diaz et al. [19] also reported similar findings. Cella et al. [20] developed a multivariate NTCP model for radiation-induced hypothyroidism including V30 and thyroid volume. In our analysis, we found a significant correlation between thyroid volume and hypothyroidism (p = 0.01). This correlation may be explained by previous surgical procedure. The incidence of hypothyroidism induced by radiotherapy depends on the type of surgical procedure and is high in patients in whom hemithyroidectomy was performed as a part of surgery. Biel et al. [23] showed that the incidence of hypothyroidism in groups of only surgery, radiotherapy and radiotherapy after surgery patients was 47%, 23% and 70%, respectively. These data have been validated by other authors [6,14,25].

The effect of age on hypothyroidism after radiotherapy is not clear. Hancock et al. [1] showed that there is a relationship between hypothyroidism and age. Previous studies by Posner [21] and Hancock [1] showed that female patients had an increased risk for hypothyroidism after radiotherapy. Vrabec et al. [22] observed that female patients had smaller thyroid reserve. However, there are some studies that have shown that there is no correlation between sex and hypothyroidism [18]. We did not find any correlation between sex and thyroid function disorder after radiotherapy in our study. We did not find any correlation between chemotherapy and comorbidity, either.

Radiation-induced hypothyroidism develops after a median interval of 1–1.8 years [21-23]. In our study, hypothyroidism developed 7–24 months after the completion of treatment (Table 2). Therefore, it may be recommended to perform a regular TSH level test from 6^th^ months follow-up after radiotherapy.

In conclusion, the clinical and dosimetric predictors of radiation induced thyroid disorders are not typically distinguished. We found a significant association between the development of hypothyroidism and the V30. Therefore, the V30 may be useful in comparing competing treatment plans to evaluate the risk of hypothyroidism when designing individual patients’ treatment.

## Competing interest

The authors declare that they have no competing interest.

## Authors’ contributions

ZA involved in the conception and design of the study; acquisition, analysis and interpretation of data; drafting the manuscript and revising it critically for important intellectual content; and giving final approval of the version to be published. BMA involved in the conception and design of the study; acquisition, analysis and interpretation of data; drafting the manuscript and revising it critically for important intellectual content; and giving final approval of the version to be published. ZO involved in the conception and design of the study, the acquisition, analysis and interpretation of data, revising the manuscript critically for important intellectual content and giving final approval of the version to be published. DY involved in the analysis and interpretation of data, revising the manuscript critically for important intellectual content; and giving final approval of the version to be published. BG involved in the analysis and interpretation of data, revising the manuscript critically for important intellectual content giving final approval of the version to be published. MS involved in the analysis and interpretation of data, revising the manuscript critically for important intellectual content and giving final approval of the version to be published. UA involved in the conception and design of the study, revising the manuscript critically for important intellectual content and giving final approval of the version to be published. All authors read and approved the final manuscript.
